# Automated Detection of Macular Diseases by Optical Coherence Tomography and Artificial Intelligence Machine Learning of Optical Coherence Tomography Images

**DOI:** 10.1155/2019/6319581

**Published:** 2019-04-09

**Authors:** Soichiro Kuwayama, Yuji Ayatsuka, Daisuke Yanagisono, Takaki Uta, Hideaki Usui, Aki Kato, Noriaki Takase, Yuichiro Ogura, Tsutomu Yasukawa

**Affiliations:** ^1^Department of Ophthalmology & Visual Science, Nagoya City University Graduate School of Medical Sciences, Nagoya, Japan; ^2^Technology Laboratory, Cresco Ltd., Tokyo, Japan

## Abstract

**Purpose:**

Although optical coherence tomography (OCT) is essential for ophthalmologists, reading of findings requires expertise. The purpose of this study is to test deep learning with image augmentation for automated detection of chorioretinal diseases.

**Methods:**

A retina specialist diagnosed 1,200 OCT images. The diagnoses involved normal eyes (*n*=570) and those with wet age-related macular degeneration (AMD) (*n*=136), diabetic retinopathy (DR) (*n*=104), epiretinal membranes (ERMs) (*n*=90), and another 19 diseases. Among them, 1,100 images were used for deep learning training, augmented to 59,400 by horizontal flipping, rotation, and translation. The remaining 100 images were used to evaluate the trained convolutional neural network (CNN) model.

**Results:**

Automated disease detection showed that the first candidate disease corresponded to the doctor's decision in 83 (83%) images and the second candidate disease in seven (7%) images. The precision and recall of the CNN model were 0.85 and 0.97 for normal eyes, 1.00 and 0.77 for wet AMD, 0.78 and 1.00 for DR, and 0.75 and 0.75 for ERMs, respectively. Some of rare diseases such as Vogt–Koyanagi–Harada disease were correctly detected by image augmentation in the CNN training.

**Conclusion:**

Automated detection of macular diseases from OCT images might be feasible using the CNN model. Image augmentation might be effective to compensate for a small image number for training.

## 1. Introduction

The major causes of legal blindness in developed countries are age-related macular degeneration (AMD), diabetic retinopathy (DR), and glaucoma [1]. In the 21st century, the development of optical coherence tomography (OCT) was a breakthrough in the ability to diagnose macular diseases and assess the necessity and efficacy of treatments [2]. Intravitreal injections of antivascular endothelial growth factor agents and sub-Tenon's or intravitreal injections of triamcinolone acetonide could not have had as great an impact on wet AMD and macular edema without the availability of OCT [3–7]. Since OCT is noninvasive and measurable even without pupillary dilation, it can be used as a screening tool. Fundus photography currently is commonly used for screening. If OCT is introduced as a screening test in addition to fundus photography, the sensitivity to detect fundus diseases will increase further. However, a retina specialist is required for precise evaluation of the OCT findings.

Artificial intelligence has been applied recently to practical engineering in face recognition systems, automated voice guidance, self-driving cars, and computer chess engines. Machine learning is a computational technology programmed to recognize patterns from a large data set. Deep learning is one of the machine learning techniques with a multilayered convolutional neural network (CNN) model to learn and detect image features [8]. Gulshan et al. reported recently that a deep learning-trained algorithm detected moderate to severe DR and referable diabetic macular edema with high sensitivity and high specificity from fundus photographs in patients with diabetes mellitus [9]. More recently, a deep learning has been applied to automated segmentation of OCT images and automated diagnosis of macular diseases [10]. Generally, the construction of an intelligent CNN model requires tremendous number of database images. One of the techniques to overcome this problem is image augmentation by a variety of image modifications such as rotation, shifting, and adjustment of magnification, contrast, and brightness.

In the current study, we evaluated the feasibility of a CNN model trained on the basis of deep learning with image augmentation by horizontal flipping, rotation, and translation for automated detection of macular diseases from OCT images.

## 2. Materials and Methods

This study was performed in accordance with the tenets of the Declaration of Helsinki. The Ethical Review Board at Nagoya City University Graduate School of Medical Sciences approved the study protocol. The study was conducted by use of an opt-out consent process and the synonymization of patient information, based on the low risk and the potential benefit for patients in this study.

### 2.1. Data Sets

OCT images obtained between 2010 and 2015 at Nagoya City University Hospital were obtained retrospectively. Patient information was synonymized and unconnected to OCT images before transfer to the study investigators. In the current study, a pair of horizontal and sagittal fovea-centered sectional images obtained by Cirrus HD-OCT, Model 4000 (Carl Zeiss Meditec AG, Jena, Germany), was used. Images that were unclear because of hazy media such as dense cataracts, fixation failures during the image capture, and other reasons were excluded. Six hundred eyes of 300 patients were chosen randomly. A total of 1,200 OCT images of 600 eyes were collected. An experienced ophthalmologist (T. Y.) provided one diagnosis to a pair of OCT images in a masked fashion. When there were multiple diagnostic suggestions, the most pathological diagnosis was recorded.

### 2.2. Training of a CNN Model

To train a CNN model, 1,100 of 1,200 OCT images were chosen randomly. Caffe, an open-source CNN framework, was used for deep learning (http://caffe.berkeleyvision.org/). A CNN model is based on cifar10_quick with three convolution and pooling layers and accompanied by a “dropout” layer. The original 1,100 images were augmented with horizontal flipping, rotation, and translation. Consequently, 59,400 images were used for deep learning. The deep learning processes were performed on an Amazon Web Service EC2 Instance with a graphics processing unit (https://aws.amazon.com/ec2/). Finally, the accuracy of the trained CNN model reached 0.85 after parameter tunings.

### 2.3. Evaluation of the Trained CNN Model

To assess the ability of the trained CNN model to accurately detect macular diseases, the remaining 100 OCT images were tested. The trained CNN model provided candidate diseases, among which normal eyes were included, with each estimated probability. The agreement between the doctor's diagnosis and candidate diseases proposed by the CNN model was evaluated. The model's precision and recall were calculated to evaluate the automated detection of macular diseases with five or more test images according to the following formulas: precision = (true positive)/([true positive] + [false positive]); recall = (sensitivity), (true positive)/([true positive] + [false negative]).

## 3. Results

### 3.1. Diagnosis of OCT Images by an Ophthalmologist

An ophthalmologist provided diagnosis for 1,200 OCT images. 570 (47.5%) images were classified as normal. The remainder were classified as follows: 136 (11.3%) as wet AMD, 104 (8.7%) as DR, 90 (7.5%) as epiretinal membranes (ERMs), 64 (5.3%) as early to intermediate AMD (drusen), 36 (3.0%) as branch retinal vein occlusion (BRVO), 32 (2.7%) as macular edema of unknown cause, 30 (2.5%) as posterior staphyloma, 28 (2.3%) as central serous chorioretinopathy (CSC), 20 (1.7%) as cystoid macular edema (CME) of unknown cause, 14 (1.2%) as macular telangiectasia type 1, 14 (1.2%) as vitreomacular traction syndrome, 10 (0.8%) as myopic choroidal neovascularization (CNV), 10 (0.8%) as dry AMD (geographic atrophy), and 42 (3.5%) as others involving macular holes, polypoidal choroidal vasculopathy (PCV), macular degeneration, inferior staphyloma, chorioretinal atrophy, Vogt–Koyanagi–Harada (VKH) disease, macular pseudoholes, central retinal vein occlusion, and unknown [11].

### 3.2. Agreement between Doctor's Diagnoses and Suggestions by the Trained CNN Model

**Figure 1:**
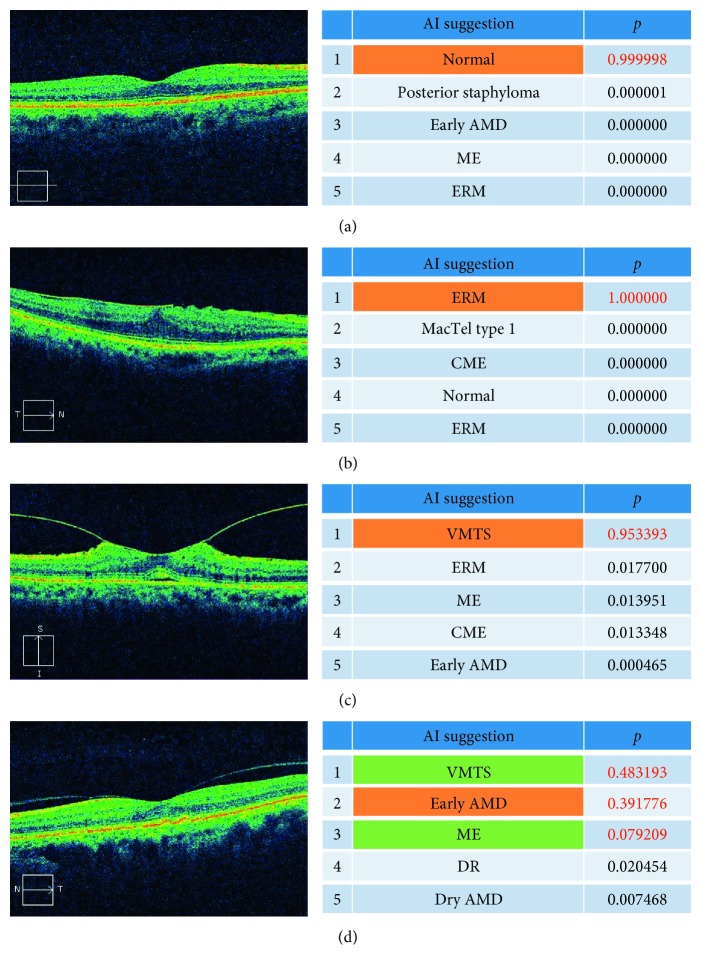
The automated top five disease suggestions and probabilities. The diagnosis is shown on each optical coherence tomography image. (a) Normal. (b) ERM. (c) VMTS. (d) Early AMD. AI = artificial intelligence; AMD = age-related macular degeneration; ME = macular edema; ERM = epiretinal membrane; MacTel = macular telangiectasia; VMTS = vitreomacular traction syndrome; DR = diabetic retinopathy; CME = cystoid macular edema.

**Figure 2:**
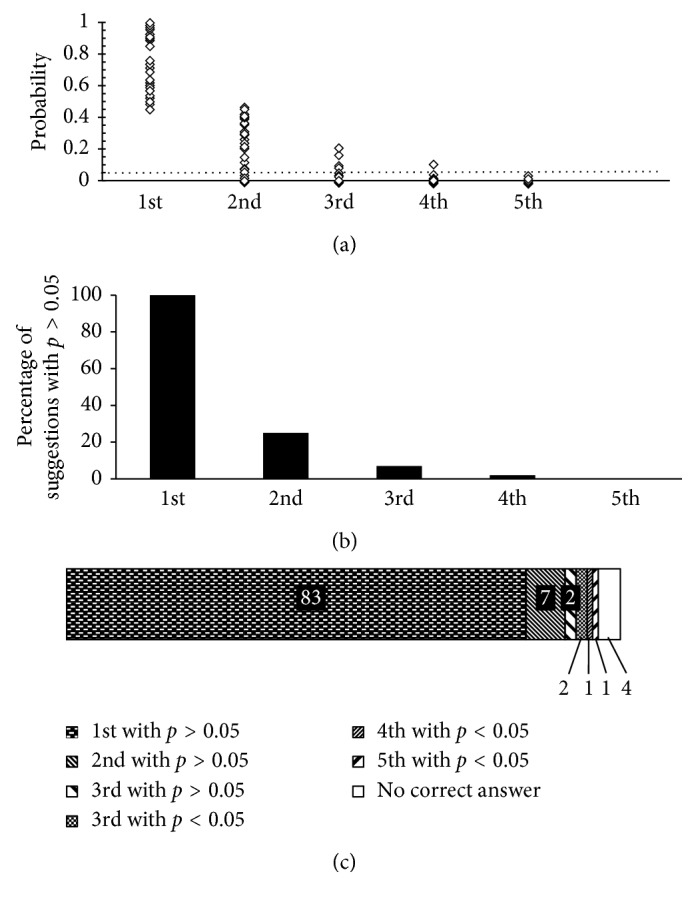
Automated suggestions of the likely macular diseases by deep learning. (a) Probabilities of the top five candidates. (b) Percentage of suggestions with a probability of 0.05 or higher in each order of candidates. (c) The order and probabilities of automated suggestions consistent with the doctor's diagnoses.

For the training of a CNN model, 1,100 of 1,200 images were randomly selected and augmented by rotation and shifting to 59,400 images. The agreement between the candidate diseases suggested by the trained CNN model and the doctor's diagnoses was evaluated.  Figure 1 shows the top five candidates in the order of probability; the normal eyes also were included. The most likely suggestions (first candidates) were correct in 83 (83.0%) of 100 images tested (reliable probability range, 0.448–1.000) (Figure 2). The second candidates were consistent with the doctor's diagnoses in seven (7.0%) images (moderate probability range, 0.147–0.450). The third candidates were correct in four (4.0%) images, two of which had a probability higher than 0.05. The fourth or fifth suggestions were the same as the doctor's diagnoses in one eye each, which did not achieve a reliable probability of 0.05 or higher. The remaining four images did not receive the correct diagnoses, i.e., one image of wet AMD had confusing findings, two images with minimal findings (CME and geographic atrophy (dry AMD)), and one horizontal image of inferior staphyloma.

### 3.3. Precision and Recall for Detection of Major Diseases

**Figure 3:**
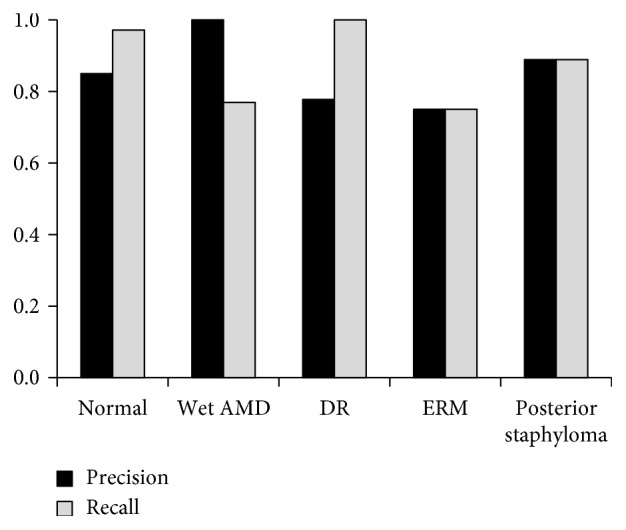
The precision and recall values of specific diagnoses. AMD = age-related macular degeneration; DR = diabetic retinopathy; ERM = epiretinal membrane.

**Figure 4:**
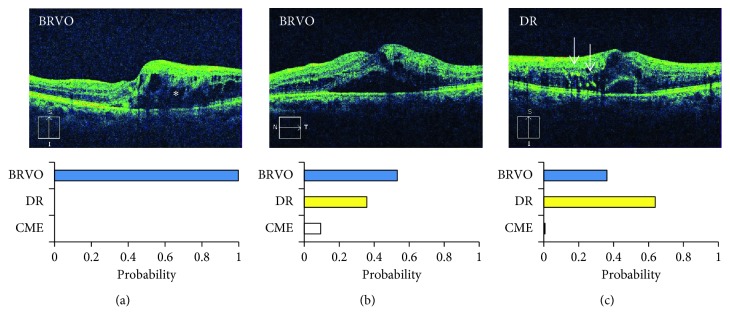
The identification of branch retinal vein occlusion (BRVO) vs. diabetic retinopathy (DR). (a) A trained convolutional neural network (CNN) identifies BRVO with a high probability possibly because of the presence of hemilateral macular edema (asterisk). (b) Bilateral macular edema might confuse the CNN. (c) Hyperreflective foci (arrows) might be a feature of DR. CME = cystoid macular edema.

**Figure 5:**
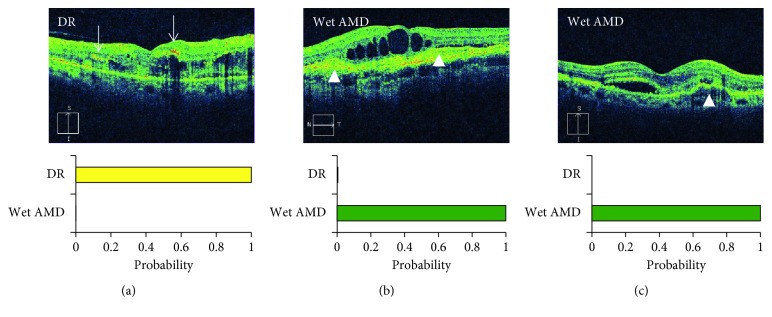
The identification of diabetic retinopathy (DR) vs. wet age-related macular degeneration (AMD). (a) The hyperreflective foci (arrows) might be a feature of DR, while (b, c) irregular elevation of the retinal pigment epithelium might be a characteristic finding of wet AMD (arrowheads).

**Figure 6:**
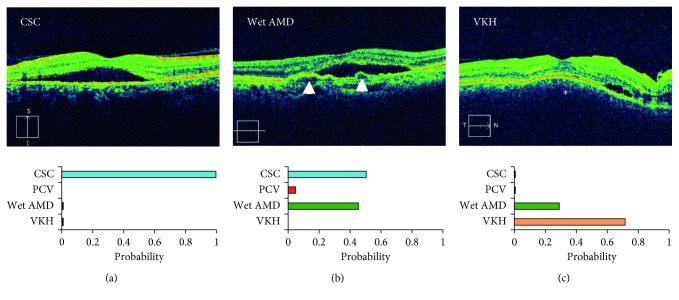
The identification of central serous chorioretinopathy (CSC), polypoidal choroidal vasculopathy, wet age-related macular degeneration (AMD), and Vogt–Koyanagi–Harada (VKH) disease. (a) A trained convolutional neural network correctly diagnoses a serous retinal detachment without elevated retinal pigment epithelium (RPE) as CSC. (b) The additional findings of irregular elevation of the RPE (arrowheads) increase the probability of wet AMD. (c) The choroidal thickening with homogeneous density (asterisk) might be a feature of VKH disease. PCV = polypoidal choroidal vasculopathy.

To evaluate the accuracy of automated detection by a trained CNN model, the precision and recall were calculated for images diagnosed as normal (*n*=35), wet AMD (*n*=13), DR (*n*=7), ERM (*n*=12), and posterior staphyloma (*n*=9) (Figure 3). The other images with other diagnoses were not assessed because of the small number of cases for each diagnosis (*n*=4 or fewer). Both the precision and recall values ranged from 0.75 to 1.00. The precision and recall were 0.85 and 0.97 for normal images, 1.00 and 0.77 for wet AMD, 0.78 and 1.00 for DR, and 0.75 and 0.75 for ERM, respectively. The CNN model guessed that six abnormal images were normal as the first suggestion; for five of those images, the second or third suggestions agreed with the doctor's diagnoses, i.e., ERM (*n*=3), early AMD with minimal elevation of the retinal pigment epithelium (RPE) (*n*=1), and macular edema with minimal changes (*n*=1). Two images of ERMs and one image of early AMD had a probability of 0.05 or higher. The diagnosis of the other image with no correct suggestions was dry AMD with local geographic atrophy. The CNN model correctly detected all seven images of DR, which suggested that the CNN model could identify characteristic findings of DR such as hyperreflective foci (Figures 4 and 5). However, the suggestion of wet AMD was precise with no false positive case, suggesting that irregular elevation of the RPE might be an identifiable feature of wet AMD and that its minimal elevation might lead to misidentification (Figures 5 and 6). ERM achieved the moderate precision and recall. This might be because some cases with a minimal change of ERM were misjudged as normal and some cases with other minimal but pathologic findings (e.g., cystoid change and irregularity of RPE) as well as ERM were judged as other diseases.

Twenty-six images had multiple suggestions with a probability of 0.05 or higher; these included images misidentified as normal or as ERMs (*n*=6), normal or early AMD (*n*=3), posterior staphyloma or myopic CNV (*n*=3), posterior staphyloma or normal (*n*=2), DR or BRVO (*n*=2), DR or CME (*n*=2), wet AMD or PCV (*n*=2), and other pairs (*n*=1 each).

## 4. Discussion

Deep learning, a technique of machine learning, is comprised of multiple processing layers for data abstraction with preservation of features. In contrast to previous machine learning techniques, deep learning can detect predictive features without computing features specified by humans. Instead, deep learning maximizes the predictive accuracy by using weight adjustment of data called back propagation [8–10]. In ophthalmology, automated evaluation of fundus photographs to detect DR has been reported previously [12–16], as has automated early detection of glaucoma based on perimetry [17].

In the current study, automated detection of macular diseases was performed by deep learning of OCT images that provided reliable suggestions with high precision and high recall. A CNN model trained on the basis of OCT images identified a variety of macular diseases involving vitreomacular traction syndrome, ERM, BRVO, DR, CSC, wet AMD, VKH disease, and posterior staphyloma. A CNN model is likely to recognize both structural changes, i.e., vitreous traction, macular holes, serous retinal detachments, and RPE detachments and exudative changes, i.e., hyperreflective foci and subretinal hemorrhages. OCT, which can visualize retinal microstructures, is currently a powerful essential tool to diagnose macular diseases and evaluate treatment efficacy. The current results showed the feasibility of applying OCT to screening examinations. For a screening examination, the precision of the suggestion of a normal eye might be critical, because low precision indicates that pathological findings might be overlooked. Nevertheless, five of six images that were wrongly suggested to be normal as the first candidate diagnosis had a correct disease suggestion in the second and third candidates and the remaining one image had a small area of geographic atrophy. Therefore, although it is ideal to improve the precision of normal images, another method to overcome this issue might be to provide the top three candidates with the estimated probability. Automated detection of macular diseases might accelerate the introduction of an OCT device into the healthcare center, which might be highly relevant from the standpoint of a health examination, nursing care, public welfare services, and remote-area healthcare.

The correct suggestions were the first and second candidates in 83 and seven of 100 images, respectively (Figure 2(c)). All 90 suggestions had a reliable probability (*P* > 0.05). The third candidate was correct in four images, two of which were reliable (*P* > 0.05). The images with agreement between the doctor's decisions and the fourth or fifth candidates could not achieve a probability of *P* > 0.05 or higher. Based on these results, the top three candidates with a probability of *P* > 0.05 or higher should be provided for disease suggestions in clinical settings. Nevertheless, the precision and recall should be improved further for ophthalmologists, especially retina specialists.

One of the major issues of this study is a small number of images for training. Previous reports dealt with a large number of database images to construct an accurate CNN model [9, 10]. The reasons involve a variety of variations of images such as individual variations of size and architecture, variations of pathologic findings (e.g., severity, location, and distribution), and variations during image capture (e.g., angle, location, magnification, blurring, and defocusing). In this study, a relatively accurate CNN model could be constructed by image augmentation by horizontal flipping, rotation, and translation. Data augmentation techniques involve horizontal and vertical flipping, rotation, outward and inward scaling, translation, invert color, Gaussian noise, the salt and pepper noise, random noise, change of contrast, brightness, color, or shape, filtering, partial masking (cutout and random erasing), trimming (random cropping), exchange background, and so on [18]. In OCT images, individual variations were small despite of age, sex, height, and race. Also, magnification and image quality are relatively stable. Therefore, major variations of OCT images might be derived from alteration of angle and location during image capture, effectively compensated for by rotation and translation of original images. Furthermore, a larger number of augmentation images by rotation <5 degrees, which may be more possible practically, and a smaller number of augmentation images by rotation <10 degrees were used in this study. In addition, because it was unnecessary to distinguish between right and left eyes in the present study, horizontal flipping was also used for image augmentation. A characteristic feature could be learned even with a small number of images sufficiently to facilitate disease identification, such as VKH disease and posterior staphyloma. On the other hand, variations of minimal pathologic findings may need a larger number of samples for training. In this study, some eyes with minimal findings such as ERMs, RPE irregularity, and retinal and chorioretinal atrophy were misidentified. A future study should determine whether these minimal changes can be detected correctly if more training images are used or other enhancing methods like transfer learning are employed [19]. Also, it is another critical issue that the CNN model cannot diagnose uncommon disorders that were not included in the original database.

Other fundamental limitations of deep learning involve the accuracy of diagnosis by retina specialists. Generally, a diagnosis should be determined from the majority of multiple doctors' decisions made in a masked manner. However, images with different decisions might have minimal, confusing findings. Actually, Gulshan et al. reported that US board-certified ophthalmologists can make decisions comparable to a trained CNN model but had considerable intergrader variability [9]. Therefore, the decisions of multiple doctors might cause variations in the decision making. A CNN model will be trained by backpropagation, concomitantly providing multiple candidate diseases with each estimated probability. Therefore, if the sample size is large, intergrader variability will not affect the training results crucially. Nevertheless, it is unclear whether the majority rule might improve the efficiency of deep learning especially in the case with small sample size for training. Inversely, the suggestions of a trained CNN model might provide unknown information, because the model might use features that are unrecognized by humans. The CNN model differentiated BRVO and DR accurately, suggesting that the model might recognize distribution of hyperreflective foci as an important feature of DR. More recently, a heatmap is available to know which area of the images is significant to provide diagnosis [19]. Thus, the unexpected abilities of deep learning might provide new insights regarding features of diseases, potentially leading to improved diagnosis and better understanding of the pathology.

## 5. Conclusions

A deep learning-based algorithm with image augmentation identified macular diseases with high precision and recall during the evaluation of OCT images. Further research should be performed to improve the accuracy of the algorithm and assess the feasibility of an artificial intelligence-equipped OCT device for screening examinations and regular examinations in clinical settings.

## Data Availability

The OCT images used for this study are restricted by the Ethical Review Board at Nagoya City University Graduate School of Medical Sciences in order to protect patient privacy. Researchers can contact Tsutomu Yasukawa, MD, PhD (yasukawa@med.nagoya-cu.ac.jp). The details of the algorithm of deep learning are available from Yuji Ayatsuka.
